# Landmarks: A solution for spatial navigation and memory experiments in virtual reality

**DOI:** 10.3758/s13428-020-01481-6

**Published:** 2020-09-16

**Authors:** Michael J. Starrett, Andrew S. McAvan, Derek J. Huffman, Jared D. Stokes, Colin T. Kyle, Dana N. Smuda, Branden S. Kolarik, Jason Laczko, Arne D. Ekstrom

**Affiliations:** 1 Department of Psychology, University of Arizona, 1503 E. University Blvd, Tucson, AZ 85721 USA; 2 Department of Psychology, Colby College, Waterville, ME USA; 3 Department of Psychology and Center for Neuroscience, University of California, Davis, CA USA; 4 Present Address: Center for Mind and Brain and MIND Institute, University of California, Davis, CA USA; 5 BrickOvenGames, Sunnyvale, CA USA; 6 Department of Psychology; Evelyn F. McKnight Brain Institute, University of Arizona, Tucson, AZ USA

**Keywords:** Spatial cognition, Navigation, Learning, Memory, Virtual reality, Unity

## Abstract

Research into the behavioral and neural correlates of spatial cognition and navigation has benefited greatly from recent advances in virtual reality (VR) technology. Devices such as head-mounted displays (HMDs) and omnidirectional treadmills provide research participants with access to a more complete range of body-based cues, which facilitate the naturalistic study of learning and memory in three-dimensional (3D) spaces. One limitation to using these technologies for research applications is that they almost ubiquitously require integration with video game development platforms, also known as game engines. While powerful, game engines do not provide an intrinsic framework for experimental design and require at least a working proficiency with the software and any associated programming languages or integrated development environments (IDEs). Here, we present a new asset package, called Landmarks, for designing and building 3D navigation experiments in the Unity game engine. Landmarks combines the ease of building drag-and-drop experiments using no code, with the flexibility of allowing users to modify existing aspects, create new content, and even contribute their work to the open-source repository via GitHub, if they so choose. Landmarks is actively maintained and is supplemented by a wiki with resources for users including links, tutorials, videos, and more. We compare several alternatives to Landmarks for building navigation experiments and 3D experiments more generally, provide an overview of the package and its structure in the context of the Unity game engine, and discuss benefits relating to the ongoing and future development of Landmarks.

One challenge in studying naturalistic behaviors, such as navigation, in the laboratory involves the three-dimensional (3D) nature of such behaviors. There are many solutions for designing two-dimensional (2D) vision-based experiments, such as Psychophysics Toolbox (Brainard, 1997), E-Prime (Psychology Software Tools, 2016), and PsychoPy (Peirce et al., 2019). Research using 3D environments, however, has largely relied on video game engines such as Unity (Unity Technologies, San Francisco, CA) to create experiments. On their own, these video game engines lack the intrinsic flexibility and features necessary for experimental design. While such flexibility and features can be developed in game engines, doing so can be extremely difficult for those without extensive programming experience or knowledge of video game design principles. While many VR hardware developers provide “asset packages” specifically for Unity that aid users in integrating the associated hardware into existing games, they still do not provide a framework for experimental design. Moreover, existing solutions for developing 3D experiments in Unity provide limited, if any, support for the growing selection of VR devices available or their associated asset packages. Here, we describe a new custom asset package for building, developing, and deploying navigation-based experiments on desktop and in VR using the Unity game engine, which is freely available for personal use. This package, called “Landmarks,” has been integrated with the Unity engine and several Unity asset packages to create a user-friendly suite of tools to facilitate the creation of VR experiments.

A game engine, such as Unity, is the ideal platform for developing navigation experiments because 3D navigation is a core component of many video games. For example, many first-person shooter video games include multiplayer modes where players can compete on a variety of maps. In addition to mastering game mechanics, the most successful players in these games often have intimate knowledge of the layout of the map they are playing on as well as how other users would use that map. Another popular style of video game involves massive open worlds that users must explore while completing tasks, quests, objectives, etc. A better understanding of the layout of the world, including important locations and landmarks, often makes the gameplay objectives easier.

Unity, even more so than other game engines, is a great option for building 3D experiments, as it provides many useful features for game development while being free for personal use. Other free game engines, such as Unreal Engine (Epic Games, Cary, NC), may provide more advanced features than Unity, but have not been available for long. Unity has extensive online documentation and support as well as a large, active user community. This is particularly helpful for developers with no formal training in video game design, as is the case for most psychologists and neuroscientists.

At the center of the Unity development platform is the Unity Editor (Fig. 1). The Editor is an integrated development environment (IDE, i.e., software for writing computer code) that provides tools to create 3D content (3D models, spatialized audio, integrated video, animation, etc.) and the ability to modify, debug, and test applications in real time. An in-depth description of Unity and the Unity Editor is beyond the scope of this paper, but a glossary of Unity terms (Unity Technologies, 2020) discussed in this paper can be found in Box 1, and an example configuration of the Unity Editor is shown in Fig. 1, featuring several often-used windows. Existing content can be used via drag-and-drop functionality across the various windows of the Unity Editor. Unity’s functionality can be extended through the use of external tools including 3D modeling software and other IDEs such as Visual Studio (Microsoft Corp., Redmond, WA). Any custom behavior in an application requires writing custom scripts using the C# programming language in an IDE, for which Visual Studio is the default in Unity.

**Fig. 1:**
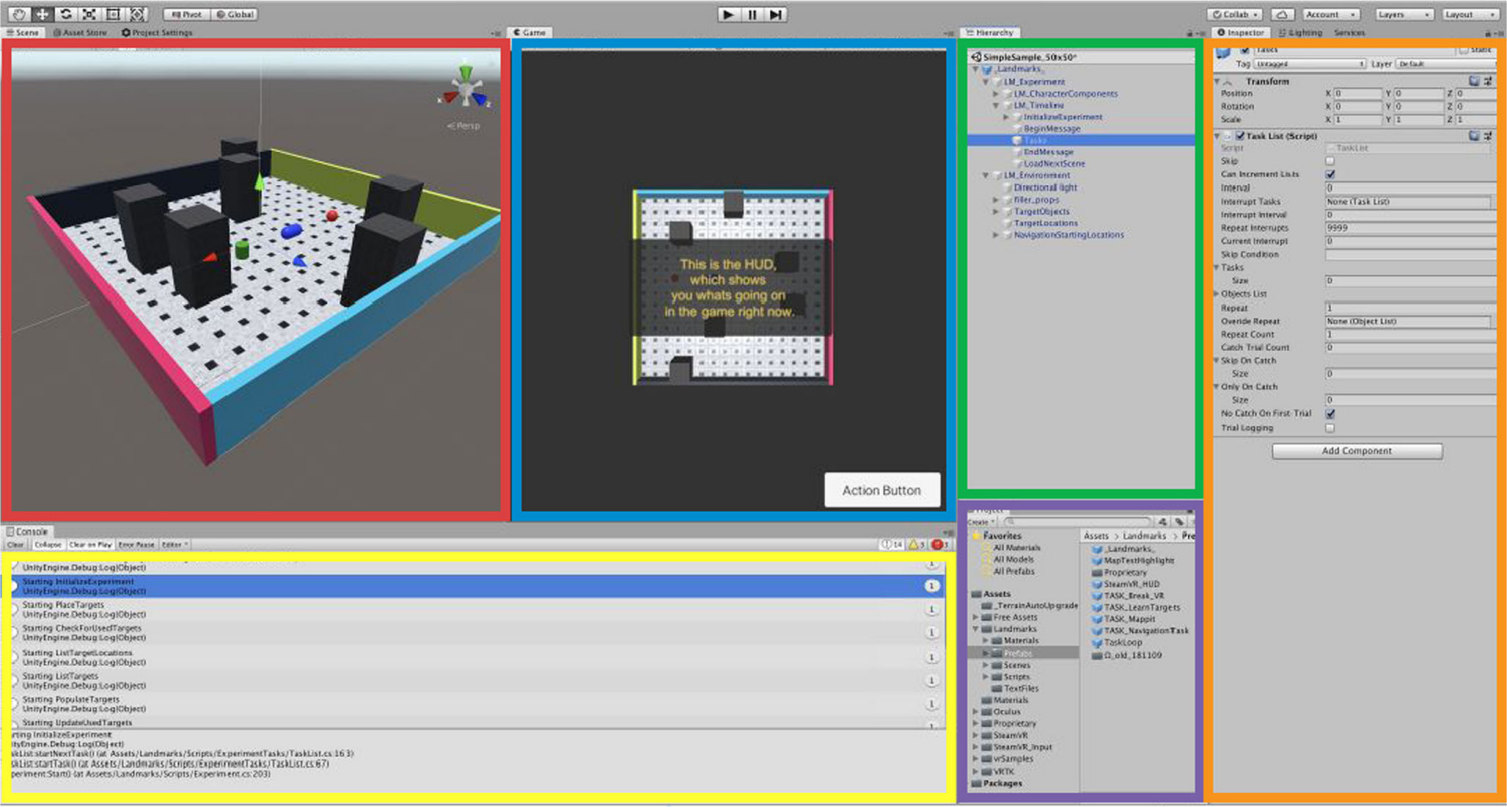
An example layout of the Unity Editor with commonly used windows highlighted (red: Scene View, blue: Game View, green: Hierarchy window, orange: Inspector window, yellow: Console, purple: Project View). A demo scene, included in Landmarks, is opened in the editor to illustrate how a user can interact with the environment and experiment via the graphical user interface of the Unity Editor rather than via code

**Box 1:** Glossary of Unity terms discussed, taken or modified from Unity’s online manual (Unity Technologies, 2020, https://docs.unity3d.com/Manual/index.html)

Unity Terms	
Asset	Any media or data that can be used in your game or Project. An Asset may come from a file created outside of Unity, such as a 3D model, an audio file or an image.
Asset Package	A collection of files and data from Unity Projects, or elements of Projects, which are compressed and stored in one file, similar to Zip files. Asset packages are a handy way of sharing and reusing Unity Projects and collections of Assets.
Camera	A component which creates an image of a particular viewpoint in your scene. The output is either drawn to the screen or captured as a texture.
Character Controller	A simple, capsule-shaped component with specialized features for behaving as a player’s avatar in a game.
Child	See *Parent*.
Component	A functional part of a GameObject. A GameObject can contain any number of components. Unity has many built-in components, and you can create your own by writing scripts.
GameObject	The fundamental object in Unity *scenes*, which can represent characters, props, scenery, cameras, waypoints, and more. A GameObject’s functionality is defined by the *Components* attached to it.
Layer	Layers in Unity can be used to selectively opt groups of GameObjects in or out of certain processes or calculations, such as being rendered by the camera.
Parent	An object that contains child objects in a hierarchy. When a GameObject is a Parent of another GameObject, the Child GameObject will move, rotate, and scale exactly as its Parent does. You can think of parenting as being like the relationship between your arms and your body; whenever your body moves, your arms also move along with it.
Prefab	An asset type that allows you to store a GameObject complete with components and properties. The prefab acts as a template from which you can create new object instances in the scene.
Project	In Unity, you use a Project to design and develop a game. A Project stores all of the files that are related to a game, such as the Asset and Scene files.
Properties	Aspects of a component that can be modified in the Inspector window of the Unity Editor.
Scene	A Scene contains the environments and menus of your game. Think of each unique Scene file as a unique level. In each Scene, you place your environments, obstacles, and decorations, essentially designing and building your game in pieces.
Tag	A reference word which you can assign to one or more GameObjects to help you identify GameObjects for scripting purposes. For example, you might define an “Edible” Tag for any item the player can eat in your game.
Texture	An image used when rendering a GameObject to give it visual detail.

Landmarks was initially commissioned by the Human Spatial Cognition (HSC) Lab as a means of reducing the amount of time required for new researchers in this laboratory to begin conducting spatial navigation experiments with Unity. The original code base was written in 2009 by Jason Laczko. This version, referred to as Landmarks 1.0 (BrickOvenGames, Sunnyside, CA), contained a basic framework of custom scripts that controlled the flow of events in Unity for experimental design and only supported desktop computer interfaces. Landmarks 1.0 was accompanied by video tutorials by Jason Laczko, which were later supplemented with additional video tutorials by Dana Smuda. Landmarks 1.0 continues to be available in its original form. This version is free to download, and tutorial videos are made available upon request.

In 2015, the HSC lab began developing experiments in virtual reality (VR) using the Oculus Rift Development Kit DK2 (Oculus VR LLC, Menlo Park, CA) and a prototype of the Cyberith Virtualizer omnidirectional treadmill (Cyberith GmbH, Vienna, AT). To incorporate these new devices into Landmarks, Michael Starrett and Jared Stokes revised the code base to provide support for both desktop and VR user interfaces. This revision was the first to utilize Git version control and was featured in an article by Starrett, Stokes, Huffman, Ferrer, and Ekstrom (2019). With the advent of the HTC Vive (HTC Corp., New Taipei City, Taiwan) head-mounted display (HMD), Landmarks required further revisions to accommodate yet another new and unique software development kit (see Huffman & Ekstrom, 2019a, b; Liang et al., 2018).

The challenge of keeping pace with emergent VR technology became evident, spurring the development of multiple character controllers such that the addition of a new controller for a new interface would not interfere with already functioning controllers. Additional revisions extended this modularization to the experimental tasks available in Landmarks and automation of settings and configurations that previously required interface- or task-specific modification by the user. This resulted in a significantly more user-friendly experience, as users can now use a single dropdown menu to select from available controllers.

Here we present a new Unity package, called Landmarks, that is bundled into a complete Unity project. Landmarks provides unprecedented integration with desktop and VR interfaces for the design of 3D experiments. Users can choose to create 3D experiments quickly and easily using drag-and-drop mechanics within the Unity Editor with no programming necessary, or they can choose to program new tasks and features on their own or alongside frequently released updates to Landmarks by using Git version control with the public Landmarks GitHub repository. In this way, Landmarks provides balance between flexible experimental design and ease of use. These features are included in the current version of Landmarks version 2.0, which is the focus of this work and will subsequently be referred to simply as Landmarks.

## Landmarks

### Software development philosophy

Landmarks is primarily maintained and developed by psychologists and cognitive neuroscientists from a range of backgrounds with varying levels of experience in programming and software development. Landmarks can be described as loosely conforming to specific, formalized software design principles. After identifying a question, or problem, experimental design often involves creating a paradigm to test hypotheses regarding that problem, piloting these paradigms, and repeating this process until the paradigm is optimized to address the question/hypothesis. Unsurprisingly, this iterative and incremental process is paralleled in the development of Landmarks.

With respect to software development, Landmarks is most consistent with an adaptive software development philosophy (Highsmith, 2013). Adaptive software development is characterized by high speed (i.e., frequent updates or releases) and high change (i.e., modifications driven largely by uncertain needs). For example, over the past year (at the time of writing) there have been over 200 (approximately four contributions per week) individual contributions to the code base on GitHub, which can be defined as any addition, deletion, or modification of code that is then uploaded to the public GitHub repository—indicative of high speed. While many of these contributions were small, many involved widespread changes including the addition of a completely new data logging system, new player controllers, and integration with third-party hardware and software—indicative of high change. This high-change and high-speed model of software development allows Landmarks to adapt to the demands of users, including staying up to date with the rapidly evolving landscape of VR hardware.

### Framework

To implement the features in Landmarks while allowing for high-change development and design, we have broken the experimental design process into four main components: Characters (cameras and player controllers; e.g., the avatar), Timeline (state machines; i.e., the sequence and structure of tasks in the experiment), Environment (3D models; e.g., streets, props, buildings), and Data (output files; e.g., text files or tab-delimited files). The first three are visually present in the implementation of Landmarks (see Hierarchy window in Fig. 1), _Landmarks_.prefab (Landmarks Prefab) Unity asset with the Experiment.cs script (Experiment) attached, and the fourth, data, is handled in the background and automatically generated for all experiments. We discuss each of these components in turn.

#### Characters

One of the most powerful features in Landmarks is that it is one of the only 3D experimental design solutions to offer compatibility with VR hardware and is, to our knowledge, the only solution that allows seamless switching between a variety of desktop and VR interface configurations. To support this functionality, a core piece of the Landmarks framework involves managing and handling various player controllers and cameras. When new player controllers are created and added to Landmarks and configured with the LM_PlayerController.cs script (LM_PlayerController), they are automatically detected and managed by Experiment.

#### Timeline

As we have mentioned, one of the greatest challenges of 3D experimental design involves incorporating the intrinsic framework of experimental design into a game engine such as Unity. Empirical research often visualizes this intrinsic framework in methods sections with figures that generally comprise a “timeline” of the experimental tasks—either pictures or descriptions of each component of the experiment ordered from left to right or top to bottom. Similarly, Landmarks implements this framework in the form of Unity GameObjects and child GameObjects, specifically the “Tasks” GameObject in the Landmarks Prefab (see Fig. 1, Hierarchy window). Just as experiments are hierarchically organized in sessions, blocks, and trials, these tasks that make up the greater experiment timeline can be set to repeat a specified number of trials and can be organized into experimental blocks and phases depending on the visual organization tasks in the form of child GameObjects of the “Tasks” GameObject. Next we will describe how these timelines and tasks function internally as finite state machines following a general structural framework.

The “Tasks” GameObject contains all of the experimental tasks that will take place in the experiment. When it comes to video game design, many of the tools rely on finite state machines to manage the flow of events (in our case, experimental tasks). In their simplest forms, finite state machines can control simple behaviors such as what happens when a light switch is flipped on or off. In this simple example, one could imagine certain aspects that could be programmed into each state (e.g., ON: any light bulb models associated with the switch begin emitting light and heat, their temperature rises, and their remaining lifespan is shortened as time passes in the on state; OFF: any light bulb models associated with the switch stop emitting light and heat, their temperature cools until it reaches baseline, and the lifespan ceases to shorten). This is only a subset of the behaviors that could be associated with these two states, and many state machines comprise a greater number of possible states.

When Landmarks is run in a functioning experiment, the Experiment finds and identifies an “LM_Timeline.” This timeline is a list of tasks defined by the associated TaskList.cs script (any object with TaskList.cs attached can be referred to as a TaskList). TaskList inherits from the base-level class defined in “ExperimentTask.cs” (any object with an associated script inherited from ExperimentTask.cs, which can be referred to as an ExperimentTask—all TaskLists are ExperimentTasks). This outlines basic experimental task states (e.g., start [what do we need to do to get ready for this task], update [what happens during this task], end [what do we need to do to close down or “clean up” after this task]), and affects operations common to all tasks that inherit from it. TaskList will hierarchically carry out any child ExperimentTask, including any other TaskLists. In summary, Landmarks implements experimental timelines via a hierarchy (i.e., GameObjects and their child GameObjects) of ExperimentTasks, with any given ExperimentTask having essentially three finite states (start, update, end). The “Tasks” GameObject is one of the child GameObjects of LM_Timeline (both are TaskLists) and represents the organization of various experimental operations that would be included in a methods figure depicting the experiment timeline in an empirical report. For example, LM_Timeline runs various ExperimentTasks such as showing a general welcome message for participants and locating and inventorying task-relevant target objects in the environment before executing “Tasks” and any child ExperimentTasks nested within “Tasks” and then closing down the experiment when there are no more ExperimentTasks to execute.

#### Environment

The third component in the implementation of Landmarks is the 3D environment. Indeed, the need for 3D environments that comprise a collection of 3D models is one of the primary reasons for utilizing the Unity game engine software in experimental design. This is largely handled through simple organization of GameObjects within the Landmarks prefab, because most of the 3D models serve only as “filler” props (i.e., task-irrelevant features of the environment), with a select few serving as experimentally crucial target objects and task-relevant locations (for either player controllers or target objects, with the latter being optional). For Landmarks to run, all users must do is provide one or more task-relevant GameObjects, with the tag “target” and the layer “targets” selected (see Box 1; for context, see Fig. 1).

#### Data

In designing Landmarks, we understand that 3D experiments are a means to an end, with that end being empirically valid data for subsequent analysis and publication. Data for any and all existing tasks are automatically handled by Landmarks via several logging scripts (dbLog.cs, LM_TrialLog.cs) that use the StreamReader and StreamWriter class of assets. Any experimentally relevant variables and values are written line-by-line to a central log file (.log extension; e.g., player position, player rotation, task state, etc., on a moment-by-moment basis) using the dblog class. Variables and values that we anticipate being immediately relevant for processing and analyzing behavioral data are added to a current trial dictionary and formatted as a row of header labels and a corresponding row of data variables; these are then logged into the central log file at the end of each trial in a task.

In addition to a central log file, which already contains all recorded data in a line-by-line temporal order, Landmarks automatically detects unique experimental tasks (logged using the LM_TrialLog class) included in an experiment, extracts this data, and formats it as a tab-delimited file (.csv extension) with a single header row, compatible with most data analysis software. Table 1 shows actual data, as organized and preprocessed, from the navigation task of the demo experiment included with Landmarks. Thus, each experiment run in Landmarks will generate a single raw data file (.log) as well as a formatted file (.csv) for each unique task included in the experiment timeline.

**Table 1:** Example data file outputted from Landmarks for a navigation task. The outputted file was opened in Microsoft Excel, pasted into Microsoft Word, and formatted based on American Psychological Association (APA) guidelines

Task	Block	Trial	Navigate_ target	Navigate_ actualPath	Navigate_ optimalPath	Navigate_ excessPath	Navigate_ duration
NavigationTask	1	1	Sphere	40.72004	13.76897	26.95107	5.892756
NavigationTask	1	2	Capsule	33.50002	34.0213	-0.521286	4.644669
NavigationTask	1	3	Cube	53.64013	51.5936	2.046532	6.048893
NavigationTask	1	4	Cylinder	36.97789	37.99688	-1.018993	4.332535

### User interaction

Every aspect of Landmarks has been designed and updated with the goal of serving the widest range of users, with an emphasis on users who have little to no programming experience. While it is virtually impossible to automate all aspects of a software package that caters to a high-change application, such as experimental design with VR integration, we wanted to provide users with a comprehensive basis of experimental design that can be used via a graphical user interface without the need for any programming—the Unity Editor. Working with Landmarks at this level involves simple drag-and-drop mechanics (place an ExperimentTask prefab in the appropriate place as a child of the experimental timeline) combined with changing values and properties of the task that are visually displayed in the Unity editor. We believe that many users will find this to be a sufficient starting point for building 3D environments and using common, simple experimental tasks from the field of navigation and spatial cognition. That said, Landmarks users are by no means restricted to the preconfigured prefab tasks that come bundled with Landmarks.

**Fig. 2:**
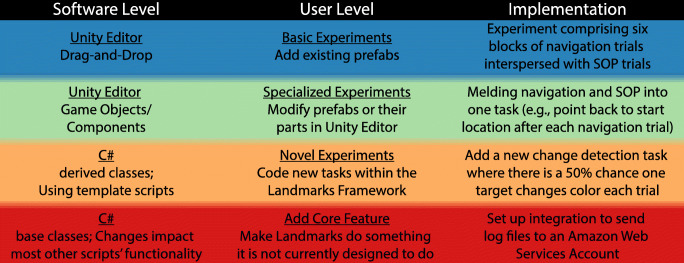
Landmarks offers a low barrier for entry-level use while maintaining a high ceiling for customizability. Each tier describes a level of software ability, use cases available at that skill level, and example implementation. Tiers are organized from least skill required (blue, top) to most skill required (red, bottom). SOP refers to the scene- and orientation-dependent pointing task, a commonly used task in spatial cognition experiments that involves participants seeing a viewpoint from an environment, orienting themselves, and pointing to a landmark

An appealing aspect of Landmarks is that users are free to “challenge by choice” (see Fig. 2). That is to say that users whose experimental designs or tasks require varying levels of complex additions or modifications to Landmarks are not necessarily “doomed” to acquire a comprehensive understanding of the C# programming language to implement such changes. Rather, Landmarks offers incremental levels of abstract use cases which require only commensurate, incrementally greater understanding of Unity and C#. Figure 2 shows four example tiers of software and programming abilities, sample descriptions for what users could accomplish at each skill level, and an example implementation in the context of a spatial cognition experiment built in Landmarks.

### The Landmarks Unity project

Landmarks is a custom asset package designed for use in the Unity game engine. Normally, adding a custom asset package to Unity would involve users obtaining a compressed file of the asset package, importing the package into the current Unity project as a custom package, and ensuring that any other asset packages required for it to work are also imported into the project. To streamline this process, Landmarks is bundled as an entire Unity project folder. This allows users to simply download the Landmarks project folder (or clone it into a local Git repository) and open it in Unity. All necessary asset packages and additional assets are already included using versions that have been tested for compatibility with Landmarks.

The primary purpose of Landmarks is to provide easy-to-use, high-quality experimental tasks and the mechanics to implement those tasks in a linear fashion consistent with experimental design and procedures. It is important to note that Landmarks alone is not sufficient to create 3D, virtual reality experiments. While Landmarks provides a user-friendly framework for implementing experimental design in Unity, many of the features and functionality built into Landmarks are only possible through the use of other open-source asset packages for Unity. These include SteamVR (Valve Corporation, Bellevue, WA), VRTK (Extend Reality Ltd, https://github.com/ExtendRealityLtd), Oculus Integration (Oculus VR, Menlo Park, CA), and other assets available in the Unity Asset Store (Unity Standard Assets, VR Samples, TextMesh Pro). The complete Unity project folder can be downloaded or cloned from GitHub (https://github.com/mjstarrett/Landmarks).

### The Landmarks Unity package

While Landmarks is made available in the form of a complete Unity project, for compatibility reasons, the Landmarks package is a custom asset package that appears as a folder in the Project View window of the Unity Editor (see Fig. 1), just as any other imported asset package would appear. Indeed, the Landmarks package folder appears with the other imported assets that come bundled with the Landmarks Unity project. Moreover, when developing Landmarks, we followed a structure of organization similar to many other Unity asset packages (subdirectories in the package folder that organize assets by their function in the Unity game engine, e.g., materials, prefabs, scenes, scripts). Several major categories used to organize the Landmarks package will be covered briefly.

#### Materials

The Unity engine uses materials to define the visual appearance of GameObjects in a scene. Materials reference assets called shaders and textures that provide information on how to wrap images around a GameObject’s geometry and subsequently render that image in the scene and game. A detailed description of Unity’s materials, shaders, and textures system is beyond the scope of this paper. This folder of the Landmarks package contains any materials, shaders, and textures that were created specifically for Landmarks. Contributors are encouraged to add any additional materials they create and configure to this folder. A set of basic color shaders (e.g., Red.mat) are included with Landmarks in a sub-directory named “base_shaders.”

#### Text files

Text files are the source for any written information that will be presented during an experiment. Out of the box, this folder will contain several text files, which are used by some of the prebuilt tasks included with Landmarks. These files use the “.txt” extension and are used to supply prewritten text to any object that supports text assets. For example, “Break_VR.txt” contains the unformatted text, “Please remove the VR headset and take a short break.” If this file is dragged onto the “message” property of the “BeginExperiment”—an InstructionsTask ExperimentTask—GameObject (see Fig. 1, Hierarchy window), the next time the experiment is run, participants will be greeted with a display that reads, “Please remove the VR headset and take a short break,” instead of the default welcome message preconfigured in the Landmarks Prefab. Thus, users can easily change the task instructions that are presented to participants by creating new text files.

#### Scripts

The functionality of Landmarks is powered by custom scripts we have written to implement the framework outlined above. Landmarks scripts are written in C#. Novice users may choose not to interact with these scripts at all or they may choose to only interact with them through the inspector window of the Unity Editor (see Fig. 1). Clicking on a GameObject reveals any scripts attached as components of that GameObject, so that basic properties (e.g., the number of times to run a navigation task) can be edited in the Unity Editor’s Inspector window (see Fig. 1). For those interested in programming or seeing the code for a component, the associated script can be opened directly from the Unity Editor (using Visual Studio or some other IDE).

In principle, configuring all of these scripts properly would require extensive documentation and troubleshooting. Fortunately, because these configurations are largely shared across experiments, Landmarks comes packaged with preconfigured GameObjects that already have all the necessary scripts attached and configured. In Unity, such GameObjects are called “prefabs,” which contain the necessary configurations of properties (i.e., variables that can be accessed within the Unity Editor) in common Landmarks scripts. Users without programming knowledge can easily modify these prefabs in the Unity Editor by selecting them in the Hierarchy window and changing their properties in the Inspector window (see Fig. 1). Users with C# programming experience can further customize and modify these prefabs and their scripted components to create new tasks (i.e., for other tests of spatial memory that are not currently implemented within Landmarks). For more information on components and requisite programming required, see Box 1 and Fig. 2. The LM_Dummy.cs (LM_Dummy) script is an example template for an ExperimentTask (see section on Framework). LM_Dummy includes comments in the code that serve to aid users to change and add public variables, or properties, as well as code for the three states of an ExperimentTask (start, update, end). LM_Dummy is an ideal candidate to examine for users looking to get started with programming their own new tasks and scripts in Landmarks.

#### Prefabs

The Unity game engine provides a convenient system for creating preconfigured GameObjects, called prefabs. These prefabs may already have scripted components attached to them as well as other GameObjects nested within them as “children” (children are objects that inherit the properties of their “parents,” objects that are higher up in the programming hierarchy; see Box 1 for more details). In addition to being prebuilt (i.e., already containing any necessary GameObjects), Unity prefabs are also preconfigured (i.e., those GameObjects have any necessary scripts already attached with all parameters and properties already defined). Unity’s prefab system also provides flexibility by allowing users to replace the preconfigured parameters and properties with their own custom values; users can even save their custom settings as their own prefab. In Landmarks, prefabs are at the core of the user-friendly framework and facilitate the drag-and-drop creation of experiments.

The only prefab required for design is the Landmarks Prefab, which contains child GameObjects that correspond to core components of the experimental design: Characters, Timeline, Environment, and Data (see section on Framework and Fig. 1). Characters are handled by selecting the desired user interface on the “LM_Experiment” userInterface property (e.g., ViveRoomspace for HTC Vive in an open room), Timeline is handled by adding any prefabs with the “TASK_” prefix or any GameObject that has an associated ExperimentTask component (see section on Framework), Environment is handled by organizing 3D models that make up the environment (e.g., a cube or a building prefab) in the “LM_Environment” child of the Landmarks Prefab, and Data is handled in the background by automated scripts that output data files (.log and .csv extensions).

#### Scenes

In Unity, a scene is often synonymous with a level in a video game (see Box 1). Scenes are saved individually in a Unity project using the “.unity” extension and can be opened in the Editor or loaded at runtime in a game. In Landmarks, a scene is the environment used for an experimental session, e.g., a 50 × 50 meter arena with colored walls or a 10 × 10 meter room with fluorescent lighting and wood flooring. The GameObjects that make up these environments are integrated into Landmarks prefabs, such that they can automatically interface with a variety of task prefabs (e.g., Landmarks-specific prefabs that facilitate specific experimental tasks, such as a navigation task or a map learning task) that users can drag and drop into the scene. This structure makes it easy to change the environment used for an experiment developed with Landmarks (e.g., replace a museum environment containing target exhibits to visit with an outdoor space containing sports equipment to collect). More scenes will be added in the future, and users are encouraged to contribute their scenes with their own customized environments. Landmarks comes with an example scene (demo_SimpleSample_50x50.unity) that demonstrates a working experiment in Landmarks. The scene comprises a Landmarks Prefab, a 50 × 50 meter environment with geometric target objects (sphere, cube, capsule, cylinder), and two experimental prefab tasks (TASK_LearnTargets.prefab and TASK_NavigationTask) configured. Users need only open the scene and click “play” in the Unity Editor to test the experiment.

#### Proprietary

Some resources that have been used with or created for Landmarks rely on proprietary software or packages that the authors cannot freely distribute. For example, an omnidirectional treadmill may require a Unity script or asset package that is only made available by the manufacturer to those who have purchased one of their treadmills. It may be the case that Landmarks has been made compatible with this treadmill, but such scripts are not useful unless a user has access to the proprietary assets. In this case, users can contact us to be given the Landmarks assets that will work with the proprietary hardware. This is because including these Landmarks assets (which we can distribute freely) in the Landmarks Unity project download without the proprietary software, code, or package they depend on (which we cannot distribute freely) would cause bugs and errors. At the time of writing, Landmarks provides support for proprietary assets and code for Cyberith’s Virtualizer line of omnidirectional treadmills, KatVR’s Katwalk omnidirectional treadmill (KatVR, Los Angeles, CA), and Tobii Pro’s VR integration for eye tracking with HTC Vive (Tobii Pro, Washington, D.C.). A complete, up-to-date list of all proprietary products and assets that are supported by or have confirmed compatibility with Landmarks is available on the Landmarks Wiki “About Landmarks” page, https://github.com/mjstarrett/Landmarks/wiki/About-Landmarks.

### Resources for using Landmarks

#### The Landmarks Wiki

Landmarks cannot, yet, completely eliminate the need for some additional learning and familiarization with designing 3D navigation experiments in Unity, but we intend to support Landmarks users through freely available resources by maintaining a public wiki. The Landmarks Wiki covers a range of topics, including setting up Landmarks with GitHub, using existing scenes to create your own experiment in minutes, using a new scene to create your own environments and experiments from scratch, citing Landmarks in manuscripts and presentations, and contributing to Landmarks using Git. This wiki serves as living documentation and reference material for Landmarks. It will be revised and updated to keep up with the development of Landmarks. The Landmarks Wiki can be found within the Landmarks repository on GitHub (https://github.com/mjstarrett/Landmarks/wiki).

#### Video tutorials

At the time of writing, an initial series of six videos have been produced and uploaded to the YouTube (YouTube LLC, San Bruno, CA) platform. The series, entitled “Getting Started with Landmarks,” serves as the most comprehensive user manual currently available and covers accessing the GitHub page to download Landmarks, building 3D environments with free 3D assets in Unity, creating drag-and-drop experiments in minutes, and more. Additional videos on more advanced use cases, discussed here, and specialized situations will be added over time. These videos are intended to allow users to follow along in order to learn general principles for using Landmarks, while encouraging users to attempt variations of what is shown. The videos are publicly available on YouTube, and the corresponding author can provide direct links upon request.

## Other solutions

We now turn to an overview of several other solutions for 3D experimental design that most closely resemble the goals of Landmarks. However, this is not meant to provide an exhaustive list. All of the solutions that we discuss are freely available or have a free option available. Our discussion will center on the features of each solution (mainly the game engine that powers it, and if virtual reality is supported) as well as how content (the experiment) is created, implemented, and analyzed by that solution.

### Virtual SILCton

Virtual SILCton (Schinazi et al., 2013; Weisberg et al., 2014; Weisberg & Newcombe, 2016) is a re-creation of the Temple University’s Ambler campus using models created in Google Sketchup (Google, LLC, Mountain View, CA), which were then imported into the Unity game engine. Virtual SILCton is administered primarily via a website portal and does not support immersive VR or other extended reality (XR). The software can be obtained from the Open Science Framework (https://osf.io/m8w24/). The primary purpose of Virtual SILCton is not to facilitate the creation of experiments, but rather as a tool to assess general navigation ability.

Virtual SILCton uses a route-learning task where various routes, and their connections, are learned. In addition, Virtual SILCton provides widely used and validated measures that can be deployed along with the route-learning task, including the Santa Barbara Sense of Direction (SBSOD) scale (Hegarty et al., 2002), the Philadelphia Spatial (Verbal) Ability Scale(s) (Hegarty et al., 2010), the Mental Rotation Task (Vandenberg & Kuse, 1978), and a modified version of the Spatial Orienting Task (Hegarty & Waller, 2004). Researchers can also opt to include free exploration, on-site pointing, map arrangement, off-site pointing, or distance estimates tasks (for descriptions of each task and recommended use, see the documentation for Virtual SILCton). While Virtual SILCton imposes limitations on the tasks that can be implemented, it is ideal as an assessment of general navigation ability and a viable solution for collecting and sharing large, standardized data sets across research groups and institutions. Virtual SILCton provides tools for data analysis in Microsoft Excel (Microsoft Corp., Redmond, WA).

### The navigational test suite: Intersections

The navigational test suite (Wiener et al., 2019) was developed in Unity and is delivered as a stand-alone application for Windows platforms, named Intersections. While the stand-alone application does not support VR, the Unity package in which it was developed is openly available from the Open Science framework (https://osf.io/u7yrh/), and thus could presumably be modified to support immersive VR or XR. Similar to Virtual SILCton, the Navigational Testing Suite is marketed primarily as a battery for assessment, with a focus on identifying navigational deficits associated with typical and atypical aging.

The navigational test suite provides users with the option of collecting data from three tasks: original, route repetition, and route retracing (for descriptions of each, see Wiener et al., 2019). All three tasks take place in a four-way intersection that serves as the virtual environment. Users can also modify the parameters of the various tasks, using the configuration files detailed in the documentation and publication, to better suit their needs. Because the project is available as a downloadable Unity project folder, it is also possible to perform more extensive customizations and modifications, but these are not discussed by the authors.

### Experiments in Virtual Environments (EVE) framework

The Experiments in Virtual Environments (EVE) framework (Grübel et al., 2017) is a Unity project designed to assist experimenters in the creation, implementation, and analysis of 3D experiments. Because the design of the experiment takes place in the Unity Editor, it is possible for users to build stand-alone applications to run their experiment on any platform. The authors note, however, that the database system used to store experimental data, a structured query language (SQL) database, is only configured to work with the Windows operating system, meaning that additional configuration may be required to store data on other platforms (e.g., macOS). Similarly, it is possible to configure EVE for immersive VR or XR, but this requires the user to configure these devices independently in Unity or externally using third-party plugins. The EVE framework is freely available on GitHub (https://github.com/cog-ethz/EVE). Of the solutions discussed, EVE is the most similar to Landmarks, focusing on the creation of new experiments that utilize 3D virtual environments.

EVE comes packaged with several ready-to-use environments, and users are able to create their own environments as well in the Unity Editor. Like Virtual SILCton, EVE also includes questionnaires such as the SBSOD, available out of the box. As with Landmarks, EVE contains several preconfigured tasks that can be dragged and dropped into an experiment. In-depth instructions can be found on the EVE GitHub page. One major difference between EVE and Landmarks is that EVE uses structured query language (SQL) commands to read and write information into a database. Landmarks, on the other hand, outputs a “.log” that can be inspected and manipulated by any text editing software, making it effectively identical to a “.txt” file. While there are many advantages to using a centralized database, SQL presents additional installation and system requirements for EVE to function out of the box, as well as a learning process for users who are not familiar with SQL or databases. EVE also includes a dedicated R package, evertools, which aids users in accessing and analyzing data stored in the SQL database.

### The Unity Experiment Framework (UXF)

Another recently developed solution is the Unity Experiment Framework (UXF; Brookes et al., 2020), a Unity project that provides the core building blocks of an experiment via a structured framework that users harness to design their experiments in Unity. The authors note that UXF is only compatible with Windows-based Unity projects. UXF is also the only solution that explicitly includes support for immersive VR HMDs out of the box. UXF is freely available on GitHub (https://github.com/immersivecognition/unity-experiment-framework).

One potential advantage of using UXF is that it was developed as a more general-purpose experiment building tool, relative to EVE and Landmarks, which were developed based largely on the research and experiments in the field of spatial cognition and navigation. One disadvantage is that UXF does not provide any resources for experiment presentation (i.e., 3D models for stimuli, environments, etc.). While the authors describe this as an advantage of UXF, it inevitably imposes the need for users to have a working proficiency in the C# programming language (although the authors do provide detailed tutorials and other resources on their website; https://immersivecognition.com) in order to generate functioning experiments, a point that the authors make clear in their documentation.

### Experiment-programming libraries (EPLs)

Python has become a widely used programming language, and several solutions have utilized the language in developing solutions for creating 3D navigation experiments. The Python Experiment-Programming Library (PyEPL; Geller et al., 2007) provides a flexible framework for more technically proficient users. Unlike Landmarks, PyEPL focuses on flexible customization through programming rather than drag-and-drop mechanics. This is an obvious disadvantage for less technically oriented users who may not be familiar with Python, or any programming language, and may struggle to access the full utility of PyEPL. PyEPL is only available for macOS, Linux, and Ubuntu (i.e., not Windows).

Another Python solution, PandaEPL (Solway et al., 2013), provides more 3D capabilities than PyEPL and utilizes the Panda3D graphics engine (Goslin & Mine, 2004) as opposed to the in-house engine used for PyEPL. Despite these added benefits over PyEPL, PandaEPL is still marketed for those proficient in the Python programming language. Although personal preference may play a role, novice users may find the Unity game engine’s extensive documentation and large user community to be an advantage over the Panda3D engine. Ultimately, the goals of PyEPL and PandaEPL are different from those of Landmarks in terms of the end users they were developed for. Additional information as well as other software from the creators of PyEPL and PandaEPL can be found online (memory.psych.upenn.edu/Software). Neither PyEPL nor PandaEPL appear to support immersive VR interfaces.

### Advantages to using Landmarks

Landmarks is a Unity project developed to excel in balancing customizability with ease of use. Landmarks provides added flexibility over options like Virtual SILCton or the navigational test suite, without including some of the complicated configurations required to use alternatives like EVE, UXF, PyEPL, or PandaEPL. Like other Unity-based solutions, Landmarks is freely available on GitHub (https://github.com/mjstarrett/Landmarks) and can be used to create stand-alone applications for many target platforms, and is presently compatible with macOS and Windows. Landmarks is also one of the only 3D solutions that provides compatibility with XR applications such as immersive VR, which utilizes HMDs and omnidirectional treadmills (UXF and EVE are the only other solution discussed that address XR compatibility, and both appear to require additional configuration by the end users to utilize this functionality). Moreover, the functionality of these devices is built into Landmarks such that users need only change an option to use a different interface (e.g., Keyboard and Mouse, HTC Vive, etc.). To our knowledge, Landmarks provides direct compatibility with more immersive VR devices, out of the box, than any other solution described, with support for more devices currently in development.

As we mentioned above, some alternatives to Landmarks include data analysis tools. For example, EVE has an associated R package, evertools. Despite the R package for Landmarks, discussed later, which is still in development, it is worth noting that many of the functions for extracting and preprocessing data in EVE are actually built directly into the data logging system in Landmarks. For example, evertools requires functions to retrieve data from the SQL database and compute performance measures from common tasks such as the judgments of relative direction (JRD) pointing task (e.g., get_participant_path_length or compute_jrd_2d_error, respectively, from https://rdrr.io/github/cog-ethz/evertools/api/). In this task, participants are asked to imagine standing at one landmark, facing another landmark, and then point to a third landmark. In contrast, Landmarks derives, formats, and saves this data automatically at runtime from the experiment application (see Table 1 for an example from a navigation task; for comparison, a Landmarks JRD task would output absolute and signed pointing errors for each JRD trial at runtime). Thus, users could load the associated data files (saved in a .csv format) into their favorite software package (e.g., Python, R, MATLAB, Excel, SPSS) for data analysis.

In summary, there are a variety of solutions available for 3D experimental design, each offering a unique framework for designing experiments and collecting empirical data. Landmarks is the only solution to offer fully interchangeable functionality for multiple, current VR interface configurations in addition to traditional desktop computer interfaces. Landmarks also has a low barrier for entry use in terms of software and programming knowledge required by users, while maintaining a high degree of flexibility and customizability at varying levels of software and programming skills. Lastly, Landmarks provides a more centralized framework to go from design to data, in that no additional tools or applications, beyond the Unity Editor, are required to obtain preformatted data ready for individual- or group-level analysis.

## Discussion

We present a novel tool for creating ecologically enriched experiments in realistic virtual environments, with a focus on spatial navigation and memory. While there are several solutions for this purpose, we believe that Landmarks provides an ideal balance between ease of use and customizability, making it useful for novice and experienced users alike in terms of the Unity engine or coding in C#. Landmarks is freely available, open-source software (https://github.com/mjstarrett/Landmarks). Out of the box, users have access to everything they need to create simple navigation experiments in minutes by dragging and dropping prefab resources into existing Unity scenes, with no coding required. Information on how to acquire, use, and even help develop Landmarks is available on the dedicated Landmarks Wiki.

### Promoting collaboration and open science

The primary purpose of Landmarks is to provide a freely available, reproducible, open-source solution for designing and implementing navigation experiments that requires no programming experience. While we will continue expanding the library of drag-and-drop tasks and refining the functionality of Landmarks, open-source software only truly evolves when users contribute their own work to the code base. By using Git as the version control system, Landmarks provides a means for users to manage their own code or even contribute their work for others to use. This may have the added benefit of reducing the number of bugs in the code by having more users actively testing and reviewing it. However, this can be a daunting task for many (especially given that a lack of computer science background is one of the primary reasons for using Landmarks in the first place).

To help make this task more manageable, the Landmarks Wiki provides information and links to resources on Git workflow models, how to fork or clone a repository, how to manage your own version of Landmarks with Git, how to update Landmarks without losing your own existing work, and how to merge your work into the current version of Landmarks to share with others. Ultimately, using Git is optional, and any user who wants to benefit from Landmarks can simply download the Unity project and begin working.

Even if users do not wish to utilize Git or contribute directly to Landmarks, simply using the package can help to promote open science. Because experimental sessions and environments are encapsulated in Unity scenes (files with the “.unity” extension), these single files can be shared and added to another instantiation of Landmarks in order to replicate procedures or reuse the environmental stimuli. An example scene containing an environment with a navigation experiment is included in the Landmarks asset package and has a file size of only 76 kilobytes.

Another way that collaboration is facilitated in Landmarks is through the ability to distribute experiments as individual, stand-alone applications that participants can download and run, with data being sent to a cloud-based server until the experimenter retrieves it. Currently, this is implemented using Microsoft Azure (Microsoft Corp., Redmond, WA) and an accompanying asset package (https://github.com/BrianPeek/AzureSamples-Unity), and functionality for other services such as Amazon Web Services (Amazon Web Services, Inc., Seattle, WA) may be implemented to the same end. While Landmarks and all packages included with it are freely available, users should note that using these cloud-based services, Azure for example, may incur charges for data storage.

### Future developments

As we continue to develop Landmarks, our main goals are to maintain and add compatibility for XR and VR interfaces and to expand the library of available experimental tasks and environments. Each time a new task is created, it will be added to the library of drag-and-droppable task prefabs (for novel tasks we may embargo this process until after publication of the new task). This process moves even more quickly with other users contributing to the source code. We outline several additional goals that we hope to achieve to improve the usability of Landmarks even beyond the design and data collection phases of research.

#### LandmarkR: An analysis package for R

Several alternatives to Landmarks offer custom analysis functions and even packages for analyzing data acquired using those solutions. The data output process for Landmarks has been configured with this in mind, and many of the operations carried out by these packages are done at runtime in Landmarks (e.g., computing error and formatting data into tab-delimited .csv files with headers). We hope to further encourage the use of Landmarks by eventually providing additional advanced analysis tools. We are in the early stages of developing a set of helper functions in the R environment (R Development Core Team, 2016) that will eventually become an open-source R-package, using the working title LandmarkR. The package will include existing functions that we have used in previous work but which have not yet been incorporated into formal R packages [e.g., permutation test to compute individual chance performance on pointing tasks (see Huffman & Ekstrom, 2019a, b) or functions to draw traversed routes from navigation over 2D images (see Figure S2 from Starrett et al., 2019)], as well as new analysis and plotting functions. As with the Landmarks Unity package, this analysis package will be freely available. Moreover, this will further enable users to easily improve code and recreate results obtained using Landmarks, in line with the open science objectives of Landmarks.

#### Navigate by Starr LITE: A stand-alone application for experimental design

Landmarks was coded with ease of use in mind, and many of the updates thus far have focused specifically on making Landmarks easier to use out of the box while maintaining users’ ability to modify and create new experiments from existing source code. However, this still requires that users download both Unity and Landmarks and then use Unity to create their experiment within the Landmarks framework. As users create and contribute to Landmarks, the library of available ready-to-use environments and drag-and-drop tasks will grow. Once Landmarks has reached a critical mass of environment-task combinations, even the need to drag and drop objects in a Unity scene may become somewhat unnecessary.

A long-term goal of Landmarks is to create a stand-alone application that accomplishes all of this behind the scenes. Ideally, users would simply run a Landmarks application (.app for macOS; .exe for Windows), which would launch a series of menus where users could select from the library of environments and then add sessions, blocks, and/or trials with tasks from the included library of Landmarks tasks. The application would then allow users to save this as an experiment that can be easily rerun the next time the application is opened (e.g., for another subject). Thus, users could create 3D navigation experiments not only without any programming, but also without even installing Unity.

It is important to note, however, that the growth of Landmarks environment and task libraries would be greatly bolstered by user contributions to the public GitHub repository or through sharing of such assets via other file sharing platforms or even email attachments to the corresponding author. The development of this stand-alone Landmarks application would only take place once Landmarks has built up a stable library of environments and/or tasks (through our own updates and additions or through the formation of a community of developers and contributors).

#### Long-term support for Landmarks

Originally written in 2009, Landmarks has already received ongoing support for over a decade from within the HSC lab under Dr. Ekstrom’s supervision (author ADE). Most recently, Landmarks has been significantly reengineered and refactored to facilitate a lower barrier for entry into designing 3D experiments in Unity. These changes were largely motivated by two goals: (1) to promote the continued development of Landmarks within the HSC lab, and (2) to prepare Landmarks to follow departing HSC lab members as they begin the next stage of their careers.

In line with the first goal, one of the authors (ASM) was hired primarily to work on future projects with Landmarks and continue development within the HSC lab. The hiring of dedicated research staff to support Landmarks development and internal use will serve to reduce the time required for new lab members (e.g., research specialists, graduate students, and postdoctoral fellows) who are not intimately familiar with Landmarks to orient themselves with Unity and begin conducting experiments. Thus, we aim to ensure that Landmarks can continue to exist at least in its current working form and expand to meet the experimental design needs of the HSC lab and other spatial cognition and navigation researchers.

The second goal aims to sow the seeds for Landmarks to expand its user base and applications to new laboratories and research questions, respectively. For example, one of the authors (DJH) is now the principal investigator of his own independent laboratory, which will continue to use and develop Landmarks internally and in conjunction with the HSC lab. Another author (MJS) has dedicated a substantial amount of his PhD work to the development of Landmarks to provide a flexible 3D experimental design solution for his postdoctoral and faculty research.

Ultimately, meeting these goals will serve to establish three initial development hubs for Landmarks (ADE via ASM, DJH, and MJS). Consistent with our aim to promote collaboration and open science, the establishment of a Git workflow has provided the foundation for long-term support. Each of these authors (ADE, via ASM, DJH, and MJS) maintains their own GitHub fork and is capable of coordinating any pull/merge requests with the master fork (MJS). This redundancy will help bolster the ability to provide long-term support to users and hopefully to increase the appeal for users to adopt Landmarks or even become a contributor.

### Is Landmarks right for my research?

We have outlined a need in the field for a comprehensive yet user-friendly solution to creating 3D virtual reality experiments. This is particularly critical at a time when VR is experiencing widespread and growing adoption in research applications. We have outlined the philosophy and framework through which Landmarks meets this need while also comparing Landmarks to a range of other solutions for 3D experimental design. Ultimately, it will remain up to the researcher to decide whether Landmarks fulfills their 3D experimental design needs, or whether another solution is right for them.

As noted in our discussion of alternative solutions, many labs already have software in place to meet the needs that Landmarks serves to address. Labs such as these, or labs that employ in-house programmers permanently or on an as-needed basis, may find little immediate benefit to using Landmarks given the at-hand expertise available in their own lab. We still encourage these users to try Landmarks, although these were not our target audience. Instead, we believe that Landmarks will be most useful to small labs who cannot fund professional in-house solutions, labs whose in-house solutions are outdated, labs who want to reallocate funds from paying professional programmers to data collection and analysis, and labs who are making their initial foray into 3D experiments or VR. For example, we hope that Landmarks will benefit labs that study memory more generally but not necessarily navigation, as we believe that VR represents a paradigm shift in how researchers in cognitive neuroscience will conduct experiments. Our hope is that the ease of getting started with Landmarks will contribute to its growth, as those initially drawn to Landmarks may be more likely to continue developing with it and encourage colleagues to do so as well.

### Concluding remarks

Our primary aim is for Landmarks to be widely implemented in VR experiments and as user-friendly as possible. Landmarks is freely available via a public repository on GitHub (https://github.com/mjstarrett/Landmarks) and contains a wiki with information and instructions for using Landmarks to design experiments (https://github.com/mjstarrett/Landmarks/wiki). Information is also available via the Human Spatial Cognition Laboratory’s website (http://humanspatialcognitionlab.org/software/), which provides links to obtain the current version of Landmarks, as well as the original release, and contact information for questions regarding Landmarks. We encourage interested parties and current users to reach out with any questions, issues, bug reports, and suggestions.
